# Comprehensive Generation of Historical Construction CAD Models from Data Provided by a Wearable Mobile Mapping System: A Case Study of the Church of Adanero (Ávila, Spain)

**DOI:** 10.3390/s22082922

**Published:** 2022-04-11

**Authors:** Manuel Rodríguez-Martín, Luis Javier Sánchez-Aparicio, Miguel Ángel Maté-González, Ángel Luis Muñoz-Nieto, Diego Gonzalez-Aguilera

**Affiliations:** 1 Department of Mechanical Engineering, Universidad de Salamanca, 49029 Zamora, Spain; ingmanuel@usal.es; 2 Department of Construction and Technology in Architecture (DCTA), Escuela T´ecnica Superior de Arquitectura de Madrid (ETSAM), Universidad Politecnica de Madrid, Av. Juan de Herrera 4, 28040 Madrid, Spain; lj.sanchez@upm.es; 3 Department of Cartographic and Land Engineering, Universidad de Salamanca, C/Hornos Caleros, 50, 05003 Ávila, Spain; mategonzalez@usal.es (M.Á.M.-G.); almuni@usal.es (Á.L.M.-N.); 4 Department of Topographic and Cartography Engineering, Escuela Técnica Superior de Ingenieros en Topografía, Geodesia y Cartografía, Universidad Politécnica de Madrid, Mercator 2, 28031 Madrid, Spain

**Keywords:** heritage, mobile mapping systems, wearable mobile mapping system, point clouds, 3D models

## Abstract

This paper presents the results of a complex three-dimensional reconstruction of the church of Nuestra Señora de la Asunción (Ávila, Spain) as an example of a successful process of verticalization from point clouds to a comprehensive computer-aided design (CAD) model. The reconstruction was carried out using the novel and advanced wearable mobile mapping system ZEB-REVO in combination with a lifting pole, in order to cover the whole geometry of the temple and, also, to model the different constructive elements. To this end, a set of good practices was followed, which allowed for passing from reality to the CAD model, such as the use of closed loops or even the use of different parametric and non-parametric strategies to capture the real geometry of the elements. As a result, this paper outlines the main guidelines for passing from point clouds to comprehensive CAD models, the former being useful for the application of smart preventive conservation processes, heritage building information models or even advanced numerical simulations.

## 1. Introduction

Nowadays, the application of wearable mobile mapping systems (WMMSes) for three-dimensional scanning of complex scenes is widely diverse: from natural environments [1,2,3], civil engineering [4,5], industrial environments [6,7], indoor spaces [8], archaeological tasks [9] to cultural heritage constructions [10,11]. 

WMMSes are novel devices for 3D reconstruction and are made up of two different groups of sensors that act systematically to provide a 3D reconstruction of the environment: the navigation and the remote sensing modules. The navigation module is based on an inertial measurement unit (IMU) and this device can sometimes also integrate a global navigation satellite system (GNSS) receiver. The remote sensing module is usually based on a 2D laser profilometer and can also integrate different types of cameras. A 2D laser profilometer is a compact laser scanning range finder that automatically rotates, allowing for a quick recording of any environment. WMMSes couple the spatial referencing provided by a position unit with the geometric data captured by a laser mapping sensor by using the so-called simultaneous localization and mapping (SLAM) approach [12].

Therefore, the WMMS devices are more efficient [13] and operational than other scanning systems, such as the terrestrial laser scanners (TLS) that work statically and require many stations to perform a complete reconstruction, although they are usually more precise. Furthermore, the WMMS is lighter than other traditional 3D laser scanner systems and the battery life is more optimal [14]. In [8], the Leica Pegasus: Backpack and the ZEB-REVO were compared with respect to a Z+F terrestrial laser scanner (TLS). Results show discrepancies at around 4 cm for the ZEB-REVO. In [15], the reader can find insight about the performance of this technology. In [6,7], the ZEB-REVO was compared with a TLS Faro system in an industrial context. The discrepancies found, despite being biased, were compatible with the performance indicated by the manufacturer.

The WMMS chosen for this article is the GeoSLAM ZEB-REVO [16]. This novel device is one of the most widespread WMMSes. A higher number of studies have applied it in different scenarios as cultural heritage documentation [17] and also for natural space management [2], underground documentation [5], disaster analysis [10] and documentation of industrial environments [6,7]. However, one of the main milestones of WMMSes is the passing from the point clouds to computer-aided design (CAD) or even building information modelling (BIM) structures, so these structured models can be applied to preventive conservation procedures or to simulate structural scenarios, among others. This strongly favours the documentation process and speeds up restoration projects because all the geometric information of the building is structured and available, as well as the possibility of extracting any type of measurement and parameter. As a result, working times are much shorter and the documentation process is more optimal. Within this context, some authors are advancing methodologies to convert point clouds into CAD or numerical models (compatible with structural analysis). One of the most recent methods for passing from the point cloud to the numerical model is the Cloud2FEM method developed by [18]. This method converts the point cloud into a solid model using voxel elements. In the same line, the authors of [19] apply a mesh and a re-topologizing of the mesh, allowing us to create as-built CAD models. Other alternatives are based on the use of reverse engineering procedures based on parametric primitives and non-parametric geometries, such as non-uniform rational basis spline (NURBS) surfaces. The generated CAD models are also useful to create new materials and constructive elements for BIM methodology, which is a collaborative work approach for the creation and the integral management of a building or construction project through a virtual model. The current BIM approaches for heritage documentation and modelling seek to acquire, manage, and integrate the building raw data into a single graphic-semantic structure. Its objective is to focus all the project information on a single digital information model created by all the agents involved in the project and its execution. BIM is the evolution of traditional design systems based on 2D drawings, as it can incorporate three-dimensional geometric information (3D), time information (4D), cost information (5D), environmental information (6D) and maintenance information (7D) [20]. As an integral tool applicable to construction, architecture and engineering design, BIM has brought numerous changes in these fields [21].

Please note that BIM methodology has been traditionally oriented to new buildings; thus, there is not a standard consensus for historical buildings (HBIM) yet [22], being an active field of research to date. This issue makes us consider some aspects as critical, such as the level of detail (LoD) or the level of information (LoI). The LoD determines the graphical and visual aspects of the building elements, such as geometry, location in the building, size or orientation, while the LoI stores relevant (i.e., non-graphical) information of the building elements, such as maintenance, inspection and supervision information, manufacturer information or additional images. In HBIM works, the objects have many details that are difficult to model with unique and non-parametric shapes [11].

This article presents a case of success based on the three-dimensional reconstruction of a historical church using a WMMS and then establishing a workflow to convert the point clouds into solid CAD models, which can be exported to any design, modelling or structural analysis software and even integrated into BIM/HBIM environments. According to this, the paper is structured in the next sections: Firstly, the material and methods used for this work will be defined. Then, the results of the virtualization process will be shown and, finally, the conclusions will be drawn.

## 2. Case Study

**Figure 1:**
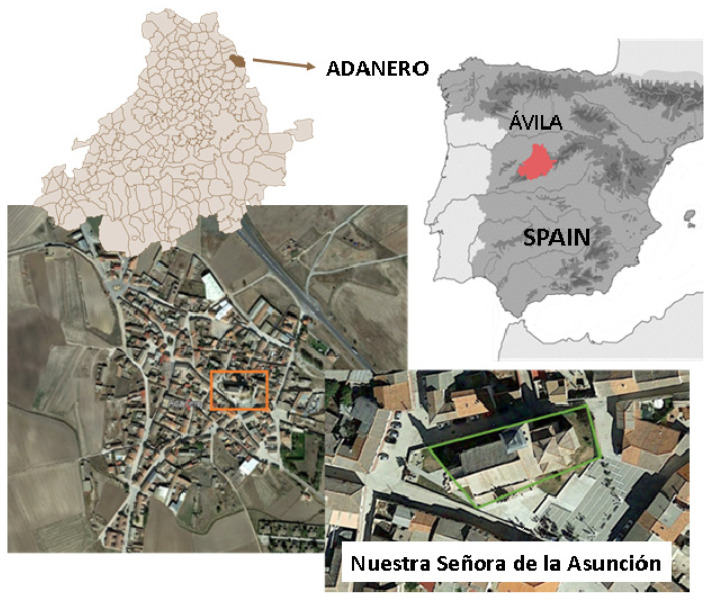
Location of the church of Nuestra Señora de la Asunción (Ávila, Spain).

**Figure 2:**
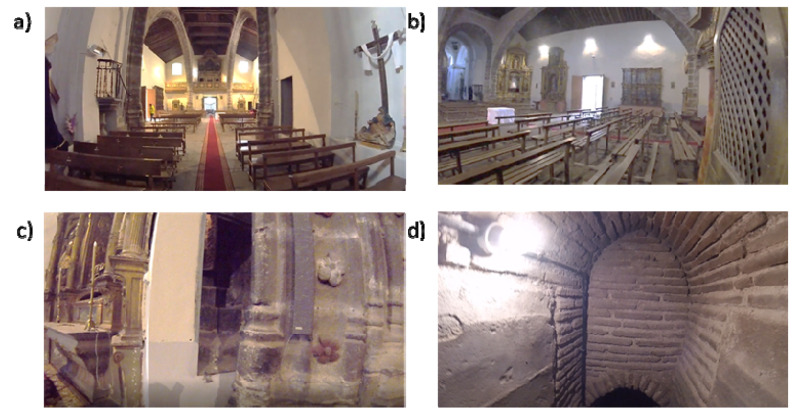
Inner views of the church: (**a**) view of the three naves from the transept of the church; (**b**) view of one of the segmental arches; (**c**) view of the entrance that gives access to the tower of the main nave; and (**d**) view of the narrow spaces that communicate the different levels of the tower.

The case study used in the present work is the church of Nuestra Señora de la Asunción. This building is located in Adanero (Ávila, Spain) (Figure 1) and dates back from the 13th century, showing a Romanesque style with Muslim influence. However, the current temple is the result of several reforms that took place in the 16th and 17th centuries. Currently, the church shows three naves. The main nave, which has a total length of 19.50 m and a total width of 7.00 m, is covered by a Mudéjar timber roof frame (Figure 2a). On the other side, the secondary naves are similar in length to the main one, showing a total width of 3.00 m. These naves are covered by timber rafters. The structural support of this timber structure was made up of two segmental masonry arches that define the three naves, as well as two masonry walls with a thickness of 0.90 m. The arches have a total span of 17.00 m, having brick masonry at their spandrel walls (Figure 2 a,b). 

**Figure 3:**
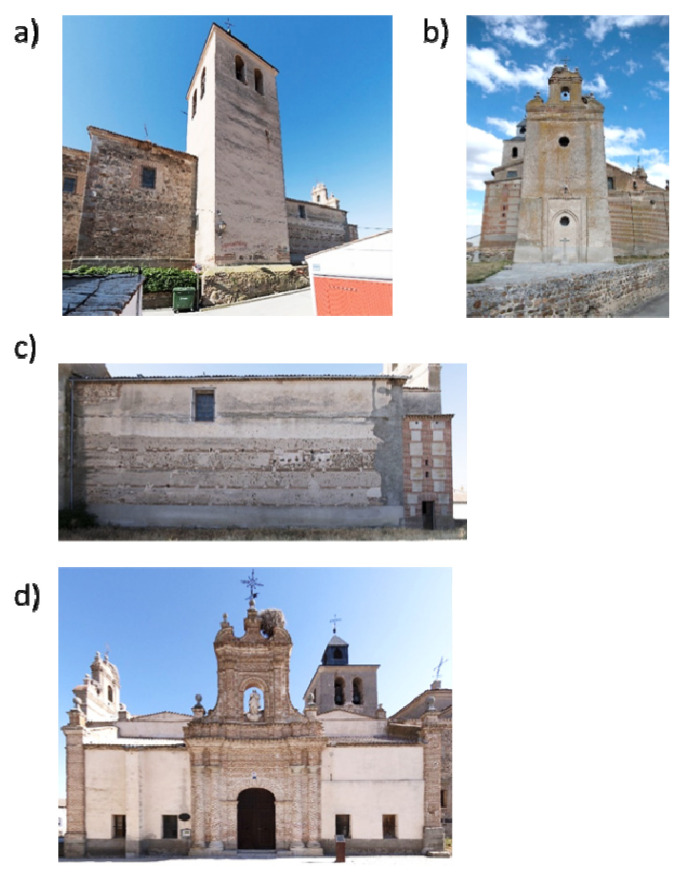
External views: (**a**) general view of the tower that is placed at the crossing of the church; (**b**) general view of the tower placed at the baptistery; (**c**) the main façade; and (**d**) opposite façade.

The current apse of the church has a Baroque style, being covered by a lunette vault. These vaults are also in the transept, having a span of 3.50 m. The cross of the church is presented by a hemispherical dome with Baroque decoration and a diameter of 6.00 m. The baptistery of the church is covered by a cloister vault with a span of 3.70 m. On this vault stands another floor, on which was located the ancient bell tower system (Figure 2a). The connection between the apse and the main nave is made up of part of the Romanesque remains (Figure 3). This space gives access to a three-story tower (Figure 2b) whose access is placed at the end of the main nave (Figure 2c). The communication between floors is solved with narrow stairs that are made up of brick masonry (Figure 2d).

Regarding its conservation status, the temple shows water infiltration problems due to the lack of maintenance of the roof. These infiltrations favour the presence of biological attacks, as well as important deformations in a great part of the rafters that are used to cover the secondary naves. Additionally, it is possible to observe out-of-plane movements in the vertical walls of these naves. All these damages demand an accurate 3D digitalization of the temple. However, the complexity of its geometry, as well as the presence of narrow spaces, hinders the use of traditional techniques such as close-range photogrammetry or static laser scanning (Figure 2c,d). Please note that close-range photogrammetry usually requires lower-cost equipment than a WMMS does; it also has the advantage of providing radiometric information. However, photogrammetry requires good light conditions (difficult in indoor spaces) as well as more time investment in terms of data acquisition and network design (i.e., overlaps, camera set-up, scales, etc.) [23]. It also involves a longer processing time (which increases with the complexity of the object to be reconstructed). On the other hand, performing three-dimensional reconstruction with a static laser scanner provides radiometric information and better geometrical accuracy but involves positioning at different stations, which greatly increases the preparation and implementation time for data acquisition. Last but not least, during data acquisition, there can be no people at the scene, so asymmetrical gross errors may appear and non-parametric approaches should be used to filter the resulting point clouds [24]. As a result, a large number of scan stations could lead to error accumulation as well as large processing times.

**Figure 4:**
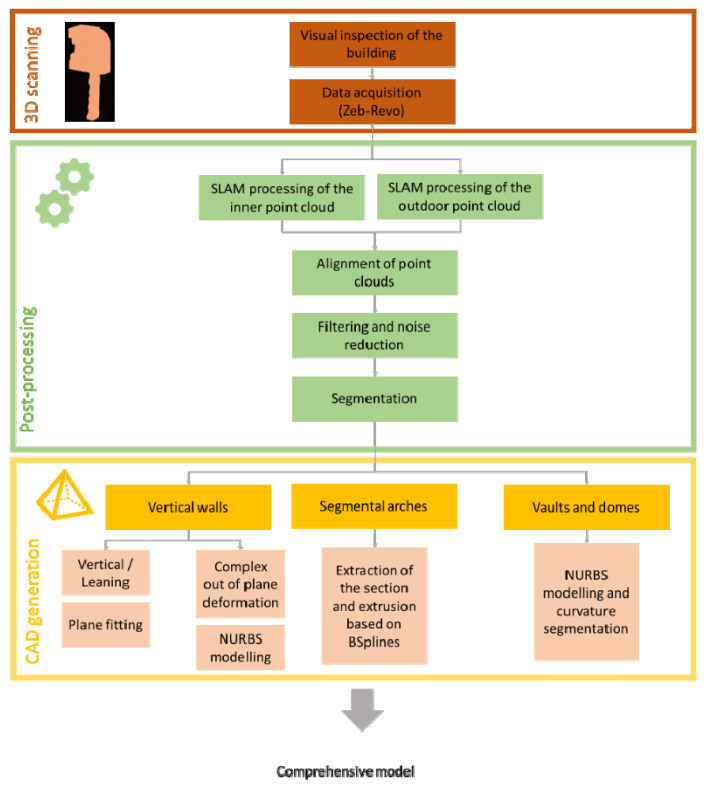
Graphical workflow used for generating a CAD model of the church.

The following methodology for digitizing the church was based on three different stages: (i) data acquisition using a WMMS; (ii) post-processing of the generated point cloud; (iii) CAD model generation (Figure 4). All these stages are described in the following sections. 

## 3. Three-Dimensional Point Cloud Generation

### 3.1. Description of the WMMS Used

The WMMS chosen for this work is the ZEB-REVO due to its versatility, the speed of data collection and the good quality of its results, validated in different previous works (e.g., [6,7,11,12]).

**Figure 5:**
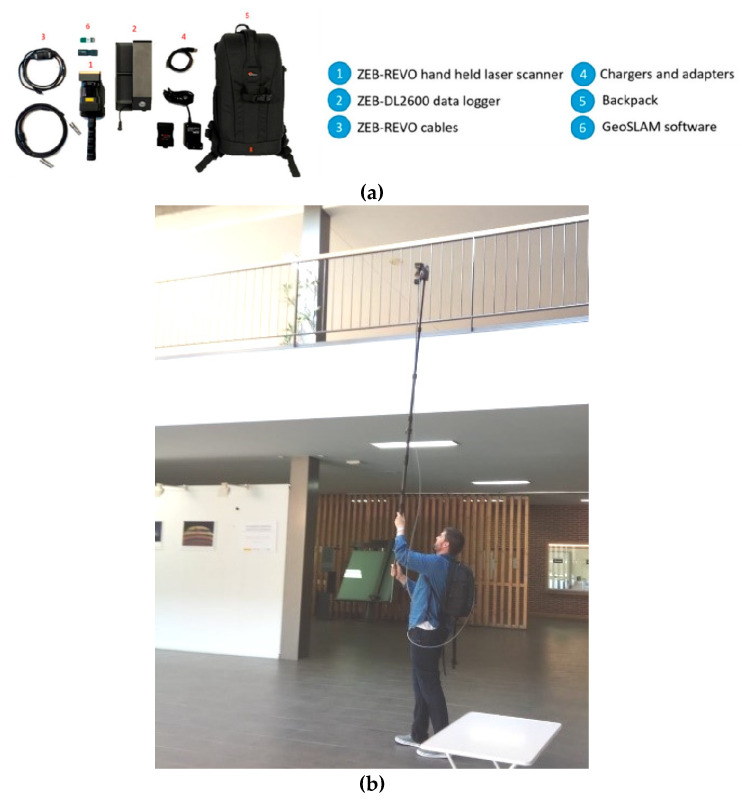
WMMS used during this work: (**a**) different technical components and (**b**) lifting pole system used for the 3D digitalization of upper parts.

The ZEB-REVO can be considered as the natural evolution of the first version, ZEBedee, which was developed by the CSIRO ICT Centre in Brisbane (Australia) [4,25]. This scanning device is based on a 2D profilometer (UTM-30LX) which is continuously rotating during data acquisition and can be displaced using platform support. Moreover, the ZEB-REVO is equipped with an IMU as a navigation module. Then, the device can be equipped with a commercial camera, the GoPro HERO, installed to record the scenario during data acquisition and, in this way, the different scanned elements can be easily identified and located in the video. This compact camera is especially useful because the ZEB-REVO does not provide radiometric information, so differentiating zones only based on geometry (without colour information) can be sometimes difficult. The integrated 2D laser profilometer is a compact laser scanner, which is more efficient, lightweight and compact than any TLS used for three-dimensional reconstruction [14]. In addition, it allows for working dynamically, eliminating the need for several static stations needed using TLS that would slow down the data collection process. Due to the continuous rotation of the sensor and the movement of the operator, three-dimensional information (i.e., points) is acquired in real time. The data are stored in a server with a hard disk that is located in a backpack, which is part of the equipment (Figure 5). Specific features of the ZEB-REVO are indicated in Table 1.

### 3.2. Acquisition Protocol

**Figure 6:**
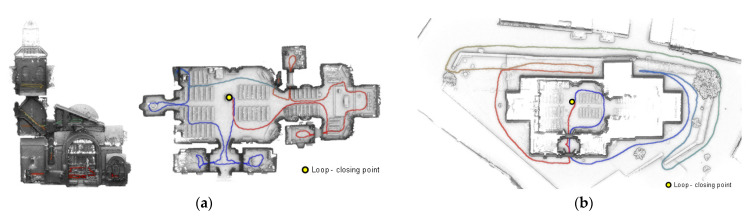
Plant representation of the different rings performed: (**a**) for capturing the indoor envelope of the church and (**b**) for capturing the external envelope. In red, the path followed, on which it is possible to observe the data captured by the GoPro camera, is represented. The overlap between rings in the central nave for the subsequent alignment should be noted.

Before data acquisition with the ZEB-REVO device, a visual inspection of the scenario was performed in order to establish the most appropriate data acquisition protocol. In accordance with this, and taking into consideration the geometry of the church with the presence of narrow spaces and a complex timber structure, it was decided to carry out a total of two closed rings (Figure 6): (i) the first for capturing the external envelope of the building and (ii) the second for capturing the inner envelope of the building. In all the rings, the protocol developed by di-Fillipo et al. [13] was applied, which could be summarized as follows: (i) ensuring accessibility to all areas, (ii) removing obstacles from the path and (iii) planning at least one closed loop to compensate for error accumulation. In addition, the walking speed was constant to ensure a homogeneous density of the point cloud.

As it was stated in the previous section, part of the digitalization was carried out by using a lift pole (Figure 5b). More specifically, we used the pole during the data acquisition carried out in rings 1 and 2. Due to this, it was possible to capture with more detail the external envelope of the church as well as its central nave. Each ring started with the initialization of the IMU on a stable and horizontal surface. Then, the laser scanner and the GoPro camera were initialized in order to capture not only the different profiles (2D point clouds) but also visual information in the form of an MP4 video. This video was referenced according to the trajectory followed during the processing stage, allowing for the spatial location of this information within the 3D point cloud. This information was useful during the visual inspection and decision process that takes place during the restoration project. The time invested was about 15 min and 19 min for the first and second rings, respectively.

The data acquired in the different rings by the laser head and the IMU were later integrated by using a full SLAM algorithm [26]. This type of SLAM algorithm allows us to process the data offline, compensating for error accumulation due to the use of closed loops. This stage was carried out by using the GeoSLAM software [16], investing: (i) 19 min for the first ring and (ii) 25 min for the second ring. For more details about the SLAM algorithm, the reader can refer to Durrant-Whyte and Bailey [26]. Subsequently, the post-processing of the point clouds was carried out through the noise reduction filters and the removal of residuals or non-significant points, considering that the noise was in the function of the laser scanner, as well as of the drift that appeared during the pose estimation implemented by the SLAM algorithm. In this work, we used the anisotropic filter proposed by Sánchez-Aparicio et al. [12]. This filter improves the accuracy of the 3D point clouds obtained by these sensors without the necessity of giving any input parameter.

**Figure 7:**
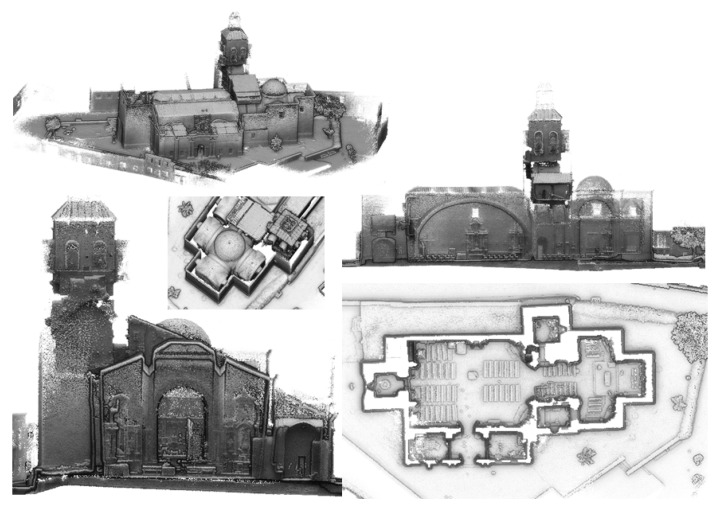
3D point cloud obtained during the digitalization process with the WMMS.

Finally, an alignment between rings was carried out, taking into consideration the overlap area captured. As a result of this, a 3D point cloud of the church was obtained. A centimetric accuracy in accordance with previous works with the same sensor and similar acquisition protocols [7,9,12] was expected (Figure 7). The whole point cloud contained a total of 42,431,420 points. 

As the reader can observe, the point clouds generated for the whole building show the geometry and details of the temple in a clear way (Figure 7). From the point clouds, the user can know the distribution of the construction, its different architectonical parts and structures, as well as the different elements installed (e.g., benches, baptismal fonts, etc.).

## 4. CAD Model Generation

The 3D digitalization of the church was performed with the aim of obtaining a comprehensive 3D CAD model of the church that supported the restoration project by means of 2D and 3D cartographic products (e.g., sections or plants). In accordance with this, it was decided to generate a CAD model with low LoD that enclosed the general geometry of the church with the exception of the timber structure and segmental arches of the naves. On the one hand, the timber elements of naves were modelled with high detail (capturing the non-linear deflections) with the aim of analysing the ultimate service state of these elements by comparing the current deflection with the maximum allowed by the Spanish code [27]. On the other hand, the high detail of the segmental arches was motivated by the necessity of evaluating the truss of these elements to the rest of the construction by performing several static graphics or limit analyses. It is worth mentioning that the assets as well as the ornamental parts of the church were not considered in this model.

**Figure 8:**
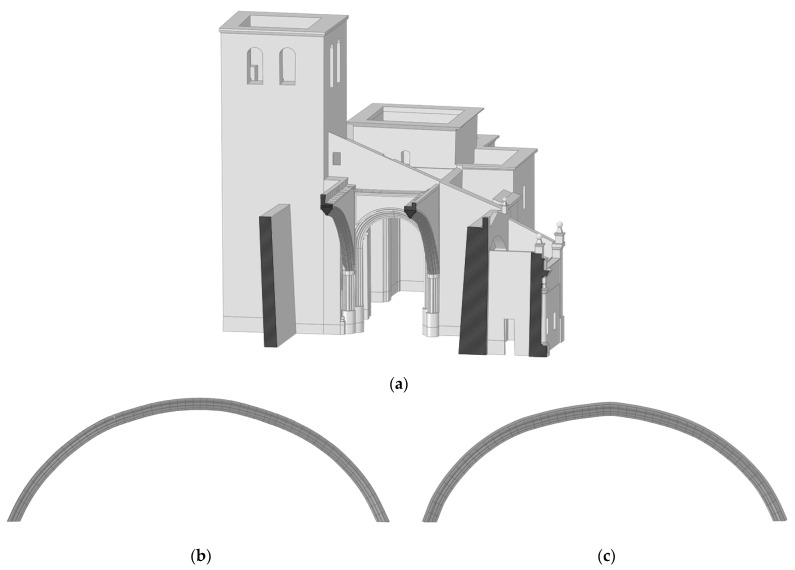
Results obtained during the modelling of the vertical walls and segmental arches: (**a**) vertical walls of the naves; (**b**) the south and (**c**) north segmental arches. It is possible to observe the ability to capture the real geometry of these constructive elements, showing inclinations and non-perfect shapes (in the case of the segmental arches).

**Figure 9:**
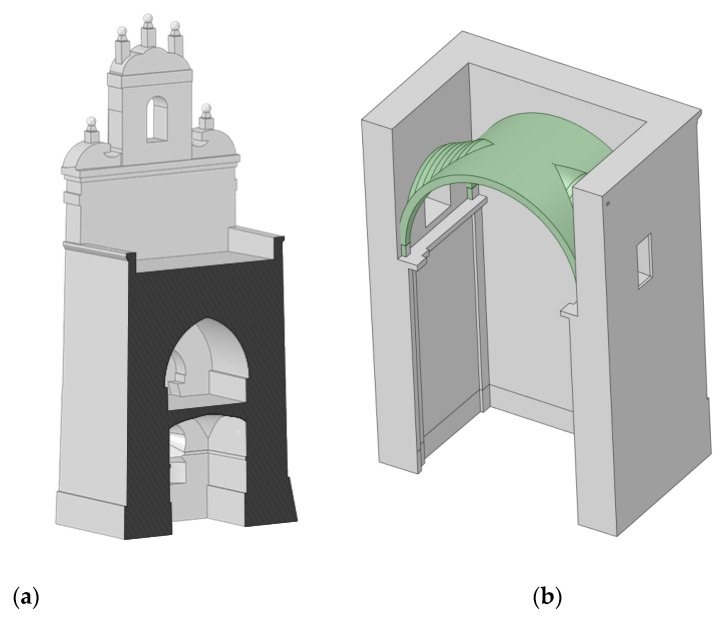
Results obtained during the modelling of different vaults: (**a**) the vaults of the tower located at the baptistery and (**b**) example of the modelling of one lunette vault at the apse of the church (the infill was not visible for clarity purposes).

**Figure 10:**
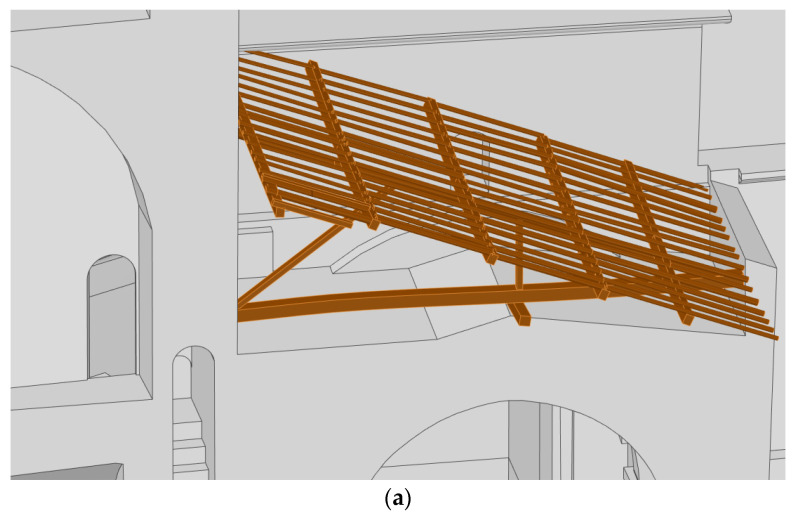

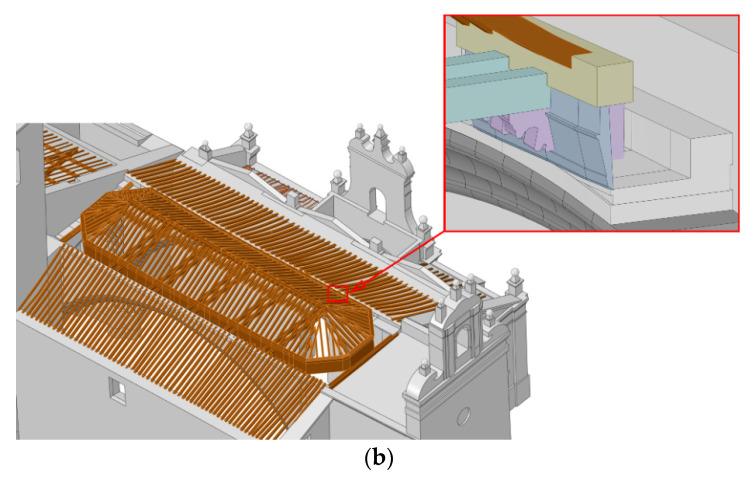
General views of the timber structure modelled in this work: (**a**) the timber elements on the transept and (**b**) the timber structure at the central nave on which it is possible to observe the high level of detail, considering all the structural elements as well as its joints.

The workflow for passing from the 3D point cloud to a CAD model is outlined in Figure 4. In this sense, we firstly segmented the point cloud into different areas, in accordance with the constructive significance (i.e., vertical walls, segmental arches, vaults, domes and timber elements) and in order to model them in a separate way. Then, a meshing stage was carried out over the segmented 3D point cloud in order to obtain a valid surface, and finally, we applied a reverse engineering procedure. This procedure was different in accordance with the type of geometry that needed to be modelled. These strategies are described in detail in the following points:

- Vertical walls (Figure 8a): These elements were modelled using the methodology proposed by Sánchez-Aparicio et al. [12]. The results classified the walls into two different groups: (i) the vertical/tilted walls [12] and (ii) the walls with complex out-of-plane deformations. The vertical/leaning walls were adjusted using the random sample consensus (RANSAC) shape detector algorithm [28], while the walls with complex deformations whose points deviated too much from the ideal fitted plane were modelled using the surface deformation approach suggested by Barrazzetti et al. [29]. This method enables complex surfaces to be modelled by a progressive adaptation of a seed surface, turning it into a NURBS surface that closely fits the point cloud. The modification of this surface was performed by changing the weights, the control points and the knot vectors of the NURBS surface until the adjustment was accomplished.
- Segmental arches (Figure 8b,c): The two segmental arches were modelled separately but using a similar procedure. Firstly, we extracted and vectorized the longitudinal trace of the arch by using a B-Spline modelling strategy. Then, we extracted and vectorized the section of the keystone with several B-Spline curves. Finally, the solid model was created by extruding the keystone section along the longitudinal trace. The brick masonry placed at the extrados of the arch was modelled by using the planes extracted after the application of the RANSAC shape detector approach. The supports of the arch were modelled by extruding the reference section (generated with B-Spline curves) in the vertical direction.
- Vaults and domes (Figure 9): The barrel vault placed at the crossing was modelled by using the deformation strategy previously shown. The arches of this vault were generated by using the strategy of the segmental arches. The vaults of the towers were modelled by defining the cross-section (B-Spline curves) and extruding it along the longitudinal direction of the vaults. The hemispherical dome was modelled by revolving the cross-section which was modelled with a B-Spline. The lunette vaults were modelled by extracting several sections in both directions and then creating loft surfaces among them. It is worth mentioning that the 3D point cloud captured by the WMMS device only covers the intrados of the elements. Thus, the application of an extra step with the aim of obtaining a solid model of these elements was necessary. In this case, the approach proposed by Sánchez-Aparicio et al. [30] was used in order to pass from the surface model to a solid model of the vaults. The thickness, infill and other stabilization elements were modelled according to with the construction rules of the epoch, as explained by Huerta Fernández [31].
- Timber elements (Figure 10): These elements, especially in the naves of the church, were modelled with high detail, including not only the deformations with high-order curves but also the joints between elements. On the one hand, the rafters of the secondary naves were modelled by extruding the vectorized section along the longitudinal trace. This trace was modelled by using a B-Spline curve. The union between these elements and the masonry walls was performed with a birdsmouth joint and a plate timber. The elements of the timber frame placed at the central nave were modelled by using the same procedure, considering the joints in [32]. The extrados of the roof was modelled by using an offset of the plane defined by the rafters, considering that the roof was built with a simple table system and ceramic tiles.

**Figure 11:**
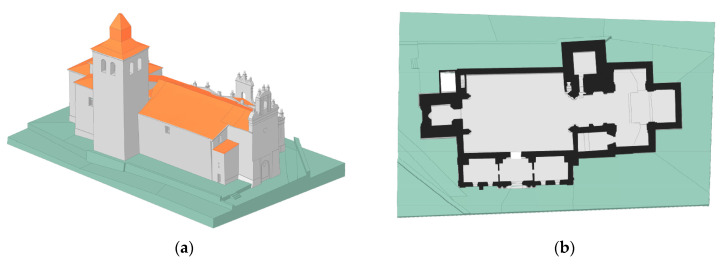

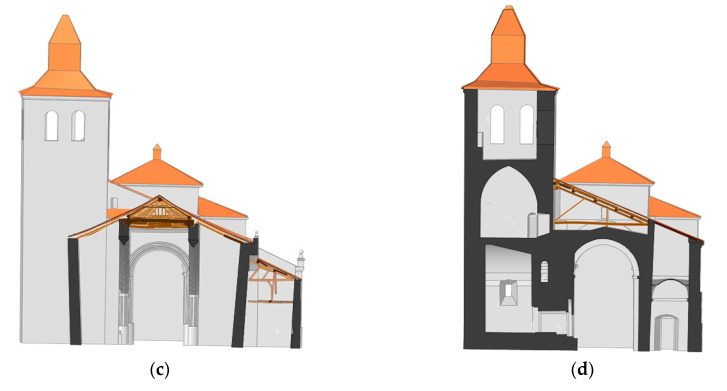
Final CAD 3D model: (**a**) general view on which it is possible to see the general geometry of the church, as well as the deformations of the roof; (**b**) plant view from the bottom to the top of the church; (**c**) cross-section along the naves; (**d**) cross-section along the tower. In grey are the masonry elements, in orange are the estimated positions of the ceramic tiles and in brown is the timber structure.

Once all the constructive elements were properly modelled, they were integrated into a unique CAD model by using Boolean operators, as well as different constructive treatises that allow us to understand the connection between parts. As a result of all these processes, it was possible to obtain a comprehensive CAD model suitable for further restoration actions (Figure 11a). In this model, the different agents could consult the geometrical aspects of the main constructive elements (i.e., the taxonomy of the archer’s voussoirs, deflection of the rafters or the general geometry of the tower) as well as extract sections and plants in different directions and heights (Figure 11b–d).

## 5. Discussion and Conclusions

The current work shows the application of WMMS technology in a challenging heritage environment, such as that of the church of Nuestra Señora de la Asuncion in Avila (Spain). Due to this device, it was possible to digitalize a great part of the building, which is made up of narrow spaces and labyrinthine rooms and towers that hinder the use of classical methods, such as structure-from-motion photogrammetry or the static laser scanner. This portable device is able to obtain a 3D point cloud of the building in about 2 h This time includes the data acquisition, SLAM processing and point cloud alignment, saving a large amount of time in comparison with the other techniques. The main disadvantage of these types of sensors is their accuracy (in the order of centimetre) according to the manufacturer’s specifications and scientific literature [7], which is significantly lower than that provided by TLS systems (in the order of millimetre), and, on the other hand, that the data provided inevitably have noise, which could make it difficult to create suitable CAD models. It implies the need to use post-processing strategies to reduce noise. In our case, we proposed the use of an efficient noise reduction filter which was tested in previous works. 

Since the full automation of 3D modelling processes does not seem to be feasible in view of the current state of the art, this paper proposes a reverse engineering pipeline to obtain the CAD model of both parametric and non-parametric shapes. As a result, a comprehensive CAD model of a complex historical construction has been generated from data taken with a WMMS as an example of the application of this type of process. This comprehensive CAD model can be used for creating as-built models and also for tasks of heritage documentation, maintenance in the context of preventive conservation and advanced numerical evaluations such as those addressed in [12]. 

## Figures and Tables

**Table 1:** Technical specifications of the WMMS used in this work.

Parameter	Value
Measurement range (indoor) (m)	30
Measurement range (outdoor) (m)	15
Data capture speed (points/s)	43,200
Accuracy	±0.1%
Relative accuracy	1–3 cm
Field of view	270° × 360°
Operating time (h)	4
Scanner dimensions (mm)	86 × 113 × 470
Weight (kg)	0.85
Rotation frequency (Hz)	0.5
